# Eltrombopag for the treatment of refractory thrombocytopenia associated with connective tissue disease

**DOI:** 10.1038/s41598-021-84493-2

**Published:** 2021-03-09

**Authors:** Juan Wang, Min Dai, Qiong Fu, Sheng Chen

**Affiliations:** Department of Rheumatology, Renji Hospital, School of Medicine, Shanghai Jiao Tong University, Shanghai, China

**Keywords:** Autoimmunity, Rheumatic diseases

## Abstract

To assess the efficacy and safety of eltrombopag in connective tissue disease (CTD)-immune thrombocytopenia (ITP), we conducted this single-center retrospective observational study, including patients with refractory CTD-ITP who were treated with eltrombopag between January 2018 and August 2019. The characteristics of patients at baseline, and the efficacy and safety of the drug were analyzed. The predictors for a response were analyzed using a univariate analysis such as Chi-square or nonparametric test and a multiple correspondence analysis (MCA) method. A total of 15 patients with refractory CTD-ITP were included in the study. Their median age at the time of inclusion was 40.6 years. The median platelet count at initiation of eltrombopag was 11.53 × 10^9^/L. The median remission time was 3.42 weeks. The complete remission (CR) and overall response rate decreased with time. The factors that associated with response to eltrombopag in patients with CTD-ITP were protopathy, WBC counts, levels of hemoglobin, and characteristics of bone marrow findings in univariate analysis. In addition, MCA indicated that a poor response to eltrombopag in patients with refractory CTD-ITP was closely associated with a protopathy with SS, medium to severe degree of anemia, leukopenia, and bone marrow aspiration showing aplastic anemia, an absence of megakaryocytes or macrophage activation syndrome (MAS). In conclusion, eltrombopag was effective and well-tolerated in patients with CTD-associated thrombocytopenia. Some factors should be considered in the use of eltrombopag, including the protopathy, blood test, and bone marrow histology.

## Introduction

Thrombocytopenia is frequent in patients with connective tissue disease (CTD). Immune thrombocytopenia (ITP) may be a primary condition or it can be associated with other disorders, such as CTD. The systemic lupus erythematosus (SLE)-associated thrombocytopenia occurs in approximately 30% of cases^1^. The primary goal of therapy for patients with chronic thrombocytopenia is to prevent severe bleeding by increasing platelets to 30 × 10^9^/L to 50 × 10^9^/L with few treatment-associated toxic effects^2^. At present, corticosteroids are used as the first-line therapy for CTD-related thrombocytopenia and second-line treatments, including immunosuppressive drugs, thrombopoietin (TPO), intravenous immunoglobulin (IVIG), splenectomy, and rituximab. Yet, not all patients respond well to this type of therapy.

Eltrombopag is an oral, small molecule, non-peptide thrombopoietin-receptor agonist that interacts with the transmembrane domain of the thrombopoietin receptor^2^. It promotes the proliferation and differentiation of megakaryocytes in bone marrow, resulting in a dose-dependent increase in normally functioning platelets^2^. Eltrombopag was approved by the FDA in 2008 for the treatment of ITP. Its efficacy and safety have been confirmed by many clinical and real-world trails in the course of the last ten years. However, so far, no clinical trials have been performed to evaluate the efficacy or safety of eltrombopag in patients with secondary ITP related to CTD. Few case reports have shown promising outcomes in SLE patients^3–5^. It showed that TPO mimetics are safe and good options for SLE-associated thrombocytopenia refractory to conventional immunosuppressive agents, and do not contribute to increased disease activity^3–5^. However, there is limited data on other CTD diseases except for SLE. Overall, more data are needed to confirm whether patients with CTD benefit from eltrombopag, taking into consideration the risk of severe adverse events, such as worsening of the underlying autoimmune dysregulation or thrombosis.

Herein, we presented 15 CTD-related thrombocytopenia cases treated with eltrombopag in Renji Hospital, Shanghai Jiao Tong University, between January 2018 and August 2019.

## Materials and methods

A total of 15 hospitalized patients with thrombocytopenia associated to CTD, including 9 patients with SLE, 3 patients with Sjögren’s syndrome (SS), and 3 other patients diagnosed with undifferentiated connective tissue disease (UCTD) in the Renji hospital, Shanghai between January 2018 and August 2019 were enrolled in the study. We confirmed that informed consent was obtained from all subjects or, if subjects are under 18, from a parent guardian. All included patients showed a poor or no response to prior high-dose steroids and immunosuppressant, such as cyclosporine A, azathioprine, and rituximab, as defining refractory thrombocytopenia associated with CTD. Inclusion criteria were: (a) subjects who fulfilled the Systemic Lupus International Collaborating Clinics Criteria (2012) for SLE^6^, American College Rheumatology (ACR), and European League Against Rheumatism criteria (EULAR) (2016) for SS^7^ or diagnosed with UCTD; (b) the baseline platelet counts ≤ 30 × 10^9^/L. Patients with arterial thrombosis, a history of venous thrombosis necessitating anticoagulation therapy, splenectomy within 12 weeks before the first screening visit, and with active malignancy were excluded from the study.

Response criteria for treatment were as follows: (a) complete remission (CR), platelet count ≥ 100 × 10^9^/L measured on 2 occasions (at least 7 days apart), and the absence of bleeding; (b) partial response(PR), platelet count ≥ 50 × 10^9^/L measured on 2 occasions (at least 7 days apart), and the absence of bleeding^8^; (c) no response, a platelet count < 50 × 10^9^/L or a less than twofold increase in platelet count from baseline or the presence of bleeding^2^. Patient characteristics and other treatments used for thrombocytopenia and their dose were also noted. The baseline platelet counts, platelet counts change curves, time to response (TTR), duration of treatment, the CR and PR rate during treatment, and adverse effects of eltrombopag were also analyzed. The predictors for a response were analyzed using a univariate analysis such as Chi-square or nonparametric test (Table 3), including age, gender, protopathy, platelet reduction time, prior therapy, anemia, white blood cells (WBCs) counts, reticulocyte counts, level of complement, bone marrow features, and so on. A multiple correspondence analysis (MCA) of the association between variables corresponds to a cross-tabulation of categorical variables and a multidimensional scaling technique. The results of MCA were graphically presented in a 2-dimensional Euclidean space. This study was approved by Renji hospital ethics committee.

### Statistical analysis

A descriptive statistical analysis was developed in Excel (Microsoft Corp). Normally and non-normally distributed continuous variables were respectively summarized as the median and interquartile range (IQR). Discrete variables were summarized as percentages. In order to identify predictors for response, we performed a univariate analysis such as Chi-square or nonparametric test of baseline variables between responder and non-responder of eltrombopag in patients with CTD-ITP. To visualize homogeneous clusters of patients with distinct response to eltrombopag, we included all baseline variables (Table 3) showing p-values < 0.1 in univariate analysis by MCA as previously described^9,10^. We stated that all methods were carried out in accordance with relevant guidelines and regulations.

## Results

### Patient characteristics

A total of 15 female patients with refractory CTD-ITP were enrolled in this study. There are 9 SLE patients, 3 SS patients, 3 patients diagnosed with UCTD. Only one patient was APS (tested positive for aPL and β2-GPI). Their median age at the time of inclusion was 40.6 years (IQR, 16–63 years). The median protopathy duration was 6.87 years (IQR, 0–31 years), the severe thrombocytopenia was 4.8 years (IQR, 0–16 years), and the median disease duration before the onset of severe thrombocytopenia was 2.07 years. In 13 patients, severe thrombocytopenia was the presenting manifestation of protopathy. The lowest platelet counts were less than 15 × 10^9^ /L in all patients. All patients were refractory to a high dose of corticosteroids (at least 1 mg/kg day) and prior immunosuppressive treatment with thrombocytopenia, including cyclosporine A (86.7%, 13/15), IVIG (53.3%, 8/15), TPO (33.3%, 5/13), rituximab (13.3%, 2/15) and other therapy (e.g., azathioprine, cyclophosphamide, mycophenolate mofetil, hydroxychloroquine). Among 15 patients with CTD-ITP, only one patient was suffering from hepatitis B cirrhosis, splenomegaly, and hyperthyroidism; 1 patient had ovarian cancer. Among 9 patients with SLE, the disease activity of one patient was severe, while most of the patients were stable or mild as measured by systemic lupus erythematosus disease activity index (SLEDAI) score.

Bone marrow aspiration was performed in 11 patients, where 5 patients showed a typical ITP. Patient characteristics, prior immunosuppressive treatments, and bone marrow feature are summarized in Table 1.

**Table 1:** Patient characteristics.

Patient	Age, years	Sex	Protopathy	Diagnosis time for protopathy	Diagnosis time for thrombocytopenia	Lowest platelet counts(× 10^9^/L)	Previous treatments	Complications	Acompanying diseases	APS	Reticulocytes ratio (%)	Bone marrow puncture	SLEDAI
1	54	F	SLE	1988	2014	2	Pred,CysA, CTX, IVIG, vincristine, transfused platelets	No	Hypertension Diabetes	Yes	2.14	NA	3
2	54	F	SLE	2005	2005	12	Pred, CysA	No	HBC,Spleno-megaly, Hyperthyroid-ism	No	NA	NA	
3	31	F	SLE	2005	2005	5	Pred, CysA, AZA	No	No	No	NA	NA	
4	47	F	UCTD	2015	2015	9	Pred, CysA	No	No	No	3.97	NA	1
5	42	F	SS	2016	2016	7	Pred, HCQ, CysA, IVIG, TPO	No	No	No	2.74	AA	
6	37	F	SLE	2013	2018	1	Pred, HCQ, CysA, MMF, IVIG, splenectomy	Autoimmune hemolytic anemia	No	No	2.31	Normal	1
7	22	F	SLE	2018	2018	9	Pred, CysA, TPO	No	No	No	1.23	ITP	5
8	16	M	SLE	2017	2017	3	Pred, CysA, IVIG	No	No	No	2.47	ITP	3
9	40	F	SLE	2018	2018	1	Pred, HCQ, CysA, IVIG, RTX	PAH	No	No	5.24	ITP	5
10	53	F	SS	2003	2003	4	Pred, HCQ, transfused platelets	No	No	No	3.39	Megakaryocytopen-ia	
11	63	M	UCTD	2019	2019	3	Pred, CysA, IVIG, TPO	No	No	No	1.73	Normal	
12	54	F	SS	2018	2018	2	Pred, CysA, IVIG, TPO	No	Ovarian cancer	No	0.55	Megakar-yocyte deficiency	
13	27	F	SLE	2014	2014	10	Pred, CysA, IVIG, CTX, RTX, plasma exchange	No	No	No	1.76	MAS	16
14	22	M	UCTD	2015	2015	10	Pred	No	No	No	NA	ITP	
15	47	F	SLE	2018	2018	12	Pred, CysA	No	No	No	NA	ITP	1

*F* female, *M* male, *SLE* systemic lupus erythematosus, *SS* sjogren's syndrome, *UCTD* undifferentiated connective tissue disease, *Pred* prednisolone, *CysA* cyclosporin A, *AZA* azathioprine, *HCQ* hydroxychloroquine, *IVIG* intravenous immunoglobulin, *MMF* mycophenolate mofetil, *TPO* thrombopoietin, *RTX* rituximab, *CTX* cyclophosphamide, *PAH* pulmonary hypertension, *APS* anticardiolipin antibody syndrome, *ITP* idiopathic thrombocytopenia, *MAS* hemophagocytic syndrome, *HBC* hepatitis B cirrhosis, *SLEDAI* systemic lupus erythematosus disease activity index, *NA* non acceptance.

### Efficacy of eltrombopag on refractory CTD-ITP

**Figure 1:**
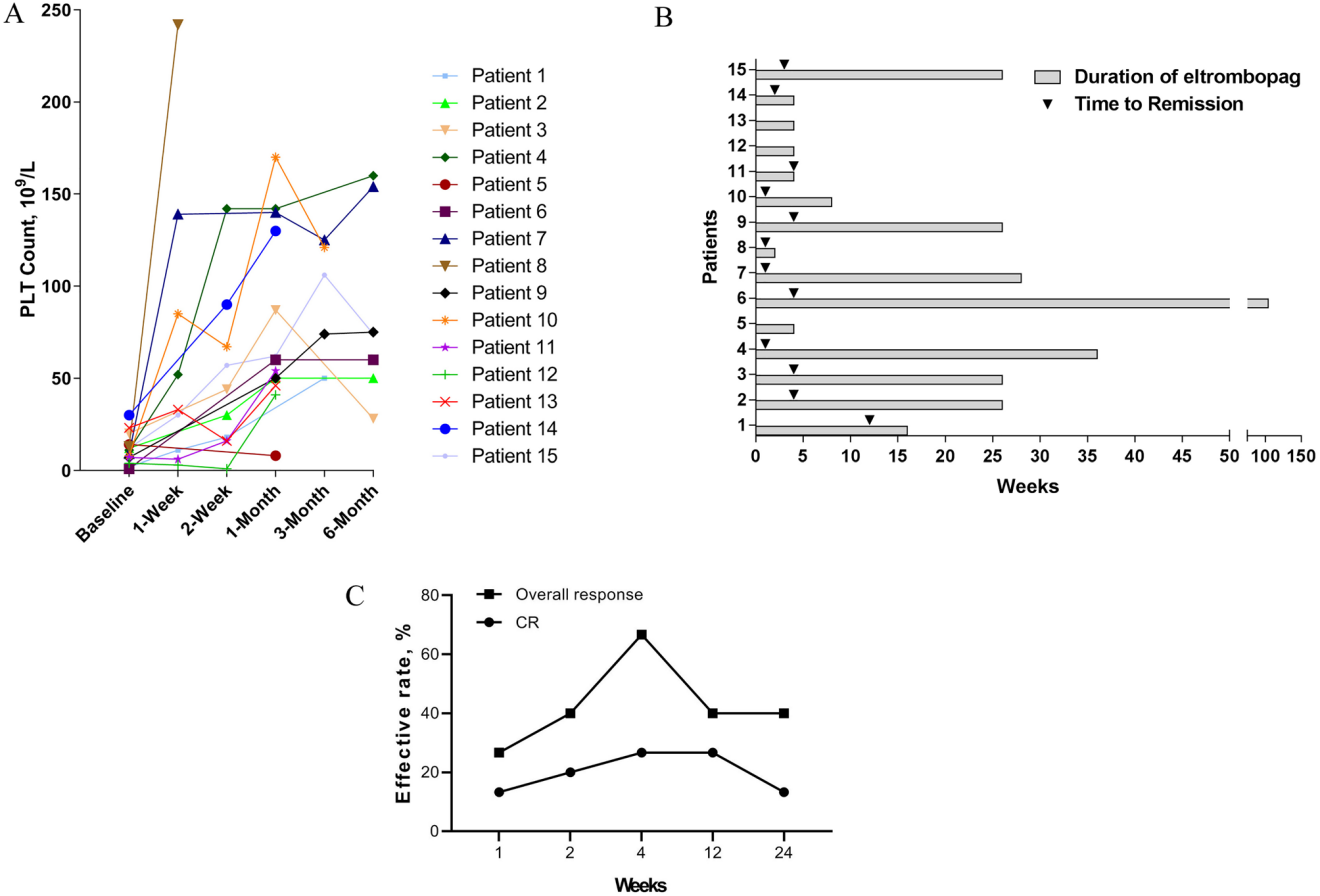
(**A**) The baseline platelet counts before treatment and platelet counts change curves at each time point during treatment with eltrombopag. (**B**) Total duration of treatment and time taken for response to therapy until August 2019. (**C**) The complete remission (CR) rate and overall response rate at each point during treatment with eltrombopag.

The median platelet count at initiation of eltrombopag was 11.53 × 10^9^/L (IQR, 1–30 × 10^9^); 12 (80%) patients had a baseline platelet count 15 × 10^9^/L or less. All subjects received eltrombopag at a dose of 25 mg/day, except one patient who received 50 mg/day. Nearly all the patients (93.33%, 14/15) completed 4 weeks treatment with eltrombopag; one patient discontinued the treatment of eltrombopag for complete remission, while 3 patients discontinued as lacking efficacy after 4 weeks treatment. The baseline platelet counts before treatment and platelet counts change curves at each time point are shown in Fig. 1A.

The median duration of therapy was 21.2 weeks (2–104 weeks), and the median remission time was 3.42 weeks (1–12 weeks) (Fig. 1B). Most of the patients (80%, 12/15) reposed well to eltrombopag. As shown in Fig. 1C, the CR and overall response rate was 26.67% and 66.67% 4 weeks later, respectively. However, the CR and overall response rate decreased with time; the CR rate was 13.33%, and the overall response rate was 40% 6 months later. The severe adverse events were seldom, except in one patient who stopped taking eltrombopag 4 weeks later for severe oral ulcers. The treatment regimen, response, TTR, duration of eltrombopag, and adverse events are shown in Table 2.

**Table 2:** Treatment regimen, response, time to response, total duration of treatment and adverse events of the drug were analysed.

Patient	Treatment regimen	Type of response (PR/CR/negative)	Time to response (weeks)	Duration of Eltrombopag, (weeks)	Adverse events
1	Pred 60 mg/day, CysA75mg/bid, Eltrombopag 25 mg/day	PR	12	16	None
2	Pred 30 mg/day, Eltrombopag 25 mg/day	PR	2	26	None
3	Pred 15 mg/day, Eltrombopag 25 mg/day	PR	2	26	None
4	Pred 40 mg/day, Eltrombopag 25 mg/day	CR	1	36	None
5	Pred 50 mg/day, CysA 50 mg/tid, Eltrombopag 25 mg/bid	Negative	–	4	None
6	Pred 60 mg/day, CysA 75 mg/bid, Eltrombopag 25 mg/day	PR	4	104	None
7	Pred 60 mg/day, Eltrombopag 25 mg/day	CR	1	28	None
8	Pred 60 mg/day, CysA 100 mg/bid, Eltrombopag 25 mg/day	CR	3 days	2	None
9	Pred 15 mg/day, Eltrombopag 25 mg/day, tacrolimus 1 mg/day	PR	4	26	None
10	Pred 40 mg/day, CysA 75 mg/bid, Eltrombopag 25 mg/day	PR	2	8	None
11	Pred 40 mg/day, CysA 75 mg/bid, Eltrombopag 25 mg/day	PR	2	4	Oral ulcer
12	Pred80mg/day, Eltrombopag 25 mg/day	Negative	–	4	None
13	Pred 40 mg/day, Tacrolimus 1 mg/day, Eltrombopag 25 mg/day, RTX 100 mg/week	Negative	–	4	None
14	Pred 5 mg/day, Eltrombopag 25 mg/day	CR	2	4	None
15	Pred 40 mg/day, CysA 75 mg/bid, Eltrombopag 25 mg/day	CR	3	26	None

*Pred* prednisolone, *CysA* cyclosporin A, *RTX* rituximab, *PR* partial response, *CR* complete remission.

### Identification of predictive factors for eltrombopag

**Figure 2:**
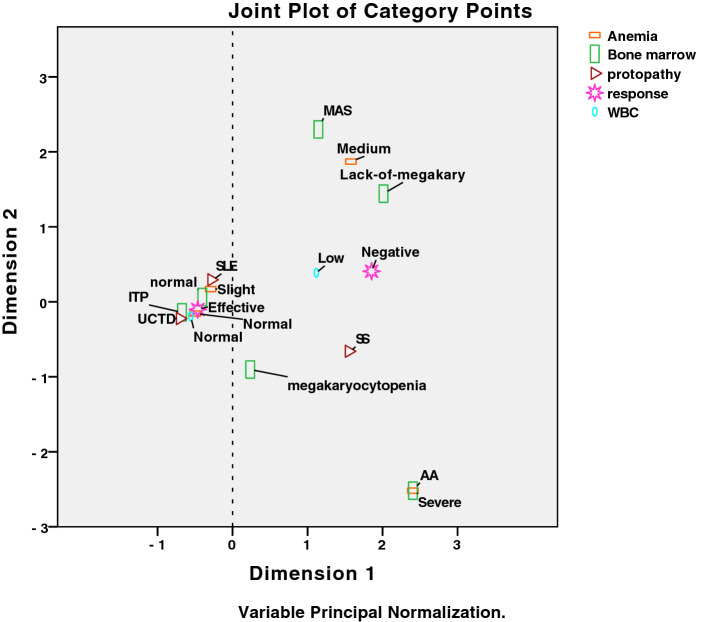
The multiple correspondence analysis (MCA) data for predictive factors for eltrombopag. Variables used in this analysis were anemia, white blood cells (WBCs) counts, bone marrow features, protopathy. For clarity, only the most discriminant variables are represented. *AA* aplastic anemia, *MAS* macrophage activation syndrome, *SLE* systemic lupus erythematosus, *SS* Sjogren's syndrome, *UCTD* undifferentiated connective tissue disease, *ITP* idiopathic thrombocytopenic purpura.

To investigate the predictive factors for eltrombopag in refractory CTD-ITP, we first compared baseline variables between responder and non-responder of eltrombopag in patients with CTD-ITP by univariate analysis. Some potentially baseline variables (defined as *p*-values < 0.1) for the efficacy of eltrombopag among patients with CTD-ITP were shown in Table 3, including protopathy (p = 0.071), WBCs counts (*p* = 0.004), levels of hemoglobin (*p* = 0.002), and characteristics of bone marrow findings (*p* = 0.01). To graphically represent the association of categorical variables in a 2-dimensional Euclidean space, we then performed a MCA based on the baseline clinical manifestations and laboratory findings. With a Cronbach's alpha of 0.905 and variance of 77.7% for the modeling, the dimension 1 (the horizontal axis) seemed to separate two distinct clusters with favorable or unfavorable factors for the use of eltrombopag in patients with refractory CTD-ITP. As shown in Fig. 2, medium to severe anemia, leukopenia, bone marrow aspiration showing aplastic anemia (AA), an absence of megakaryocytes or macrophage activation syndrome (MAS), and a protopathy with SS have been associated with the poor response to eltrombopag in patients with refractory CTD-ITP. While, patients with normal WBCs counts, no or slight anemia, and relative normal bone marrow aspiration have been associated with a good response to eltrombopag.

**Table 3:** Comparison of baseline variables between responder and non-responder of eltrombopag in patients with connective tissue disease-associated immune thrombocytopenia (CTD-ITP).

Baseline variables	Efficacy of eltrombopag		p*
	Responder, n = 12	Non-responder, n = 3	
Age, years	40.5 ± 15.1	41 ± 13.5	0.945
≥ 50 old-year	4	1	0.758
Female: Male	9:3	3:0	0.484
**Protopathy**			0.071
Systemic lupus erythematosus	8	1	0.341
Sjögren’s syndrome	1	2	0.081
UCTD	3	0	0.484
Duration of platelet reduction > 1 year	6	2	0.554
Splenomegaly	3	0	0.453
**Prior therapy**			
High dose of corticosteroids^#^	12	3	1.000
Intravenous immunoglobulin	5	2	0.446
Use of TPO	3	2	0.242
Rituximab	1	1	0.371
CysA	9	3	0.484
HCQ	4	1	0.758
CTX	1	1	0.371
**Laboratory examination**			
WBCs counts, 10^9^/L	8.0 ± 3.9	2.1 ± 0.7	0.004
Leukopenia (< 4.0 × 10^9^/L)	2	3	0.022
Hemoglobin, mg/dL	131.7 ± 25.7	70.3 ± 13.2	0.009
**Anemia**			0.002
Normal (≥ 120 mg/dL)	10	0	
Slight degree (≥ 90 mg/dL)	2	0	
Medium degree (60–90 mg/dL)	0	2	
Severe degree (< 60 mg/dL)	0	1	
Elevated reticulocyte counts	7	2	0.491
Low level of complement	6	2	0.615
**Characteristics of bone marrow findings**			0.01
Normal	6	0	
ITP	5	0	
Aplastic anemia	0	1	
Megakaryocytopenia	1	0	
Lack of megakaryocytes	0	1	
Macrophage activation syndrome	0	1	

Continuous variables were presented as mean ± (standard deviation).

*CysA* cyclosporine A, *CTX* cyclophosphamide, *HCQ* hydroxychloroquine, *ITP* idiopathic thrombocytopenic purpura, *UCTD* undifferentiated connective tissue disease, *WBCs* white blood cells, *TPO* thrombopoietin.

*p was shown with exact significance on one-sided or asymptotic significance on two-sided.

^#^High dose of corticosteroids were defined as at least 1 mg/kg day; responder, complete remission plus partial response.

## Discussion

Eltrombopag is a thrombopoietin-receptor agonist (TPO-RA) that mimics the action of endogenous TPO on megakaryocytes and megakaryocyte precursors, promoting their growth and differentiation, thus, increasing platelet production^11^. In 2018, guidelines for ITP recommended TPO-RAs for patients who did not respond well to glucocorticoids or rituximab, or those with splenectomy^12^. In this study, we performed a real-world investigation of eltrombopag treatment for patients with CTD-ITP. Our results demonstrated that eltrombopag might be an effective therapy for patients with ITP secondary to autoimmune diseases who did not respond well to standard ITP treatment.

In this study, 80% of patients responded well to eltrombopag. Most of our patients received a dose of eltrombopag of 25 mg/day, which has been recommended for patients of East Asian descent. Eltrombopag was well tolerated, except for severe oral ulcers, which were observed in one patient. The median time taken for initial response to eltrombopag was 3.42 weeks, and peak response rate occurred after a one-month treatment. Many positive results in terms of effectiveness and maintained platelet response rate of eltrombopag have been reported in the treatment of chronic ITP^13,14^. Interestingly, we found that the response rate gradually declined until reaching the plateau stage. It might be related to the small dose of eltrombopag administrated on the CTD-ITP patients, and also the clinical severity of the primary disease for CTD-ITP. Indeed, several clinical trials observed the similar plateau phenomenon in more than half of robust responders of eltrombopag in refractory severe aplastic anemia^15^ or patients with chronic immune thrombocytopenia^16^. Furthermore, the effect of eltrombopag on cell proliferation rates may also have a plateau phenomenon^17^.

A number of studies have indicated that TPO-RAs is associated with thrombotic risk when treating patients with chronic liver disease and those having antiphospholipid antibodies (aPL)^18–22^. In our study, one patient had hepatitis B cirrhosis, and another one was aPL and β2-GPI positive; yet, no thromboembolic events were observed after 26 weeks and 16 weeks treatments, respectively.

Many ITP real-world studies evaluated clinical and laboratory variables associated with response to eltrombopag and the relative factors, including age, anemia, bone marrow features, and so on^11^. In our study, we found that anemia, decreased WBCs counts, bone marrow aspiration showing aplastic anemia, an absence of megakaryocytes or macrophage activation syndrome (MAS) were predictors of worse outcome. Bone marrow characteristics such as megakaryocytopenia, bone marrow hypocellularity, and dyserythropoiesis were predictors of worse outcome^23^. Eltrombopag is effective in ITP, aplastic anemia (AA), and myelodysplastic syndromes (MDS), which is probably due to a sort of spillover specificity on other growth factor receptors, as well as to the action at the stem-cell level^11^. On the whole, although responses are observed in MDS and AA, eltrombopag is more effective in classic ITP, confirming the importance of megakaryocytic reservoir^23,24^. Indeed, marrow megakaryocyte count could be a response predictor of severe thrombocytopenia in patients with SLE and SS^25,26^. However, more prospective studies are warranted to address whether SS-ITP patients have a poor response to eltrombopag than other CTD-ITP patients.

This study has a few limitations. First, the sample size was small. Second, because this is a retrospective study, possible recall and selection errors cannot be excluded.

## Conclusions

Eltrombopag is effective and well-tolerated in CTD-associated thrombocytopenia. Some factors should be considered in the use of eltrombopag, including the disease of protopathy, blood test, and bone marrow aspiration. Additional large studies would be needed to confirm the results.
